# Local Bacteriophage Delivery for Treatment and Prevention of Bacterial Infections

**DOI:** 10.3389/fmicb.2020.538060

**Published:** 2020-09-18

**Authors:** Stijn Gerard Rotman, Eric Sumrall, Reihane Ziadlou, Dirk W. Grijpma, Robert Geoff Richards, David Eglin, Thomas Fintan Moriarty

**Affiliations:** ^1^AO Research Institute Davos, AO Foundation, Davos, Switzerland; ^2^MIRA Institute for Biomedical Engineering and Technical Medicine, Department of Biomaterials Science and Technology, University of Twente, Enschede, Netherlands; ^3^Department of Biomedical Engineering, Faculty of Medicine, University of Basel, Basel, Switzerland

**Keywords:** bacteriophage, local delivery, sustained release, infection, hydrogel, beads, embedding, encapsulation

## Abstract

As viruses with high specificity for their bacterial hosts, bacteriophages (phages) are an attractive means to eradicate bacteria, and their potential has been recognized by a broad range of industries. Against a background of increasing rates of antibiotic resistance in pathogenic bacteria, bacteriophages have received much attention as a possible “last-resort” strategy to treat infections. The use of bacteriophages in human patients is limited by their sensitivity to acidic pH, enzymatic attack and short serum half-life. Loading phage within a biomaterial can shield the incorporated phage against many of these harmful environmental factors, and in addition, provide controlled release for prolonged therapeutic activity. In this review, we assess the different classes of biomaterials (i.e., biopolymers, synthetic polymers, and ceramics) that have been used for phage delivery and describe the processing methodologies that are compatible with phage embedding or encapsulation. We also elaborate on the clinical or pre-clinical data generated using these materials. While a primary focus is placed on the application of phage-loaded materials for treatment of infection, we also include studies from other translatable fields such as food preservation and animal husbandry. Finally, we summarize trends in the literature and identify current barriers that currently prevent clinical application of phage-loaded biomaterials.

## Graphical Abstract

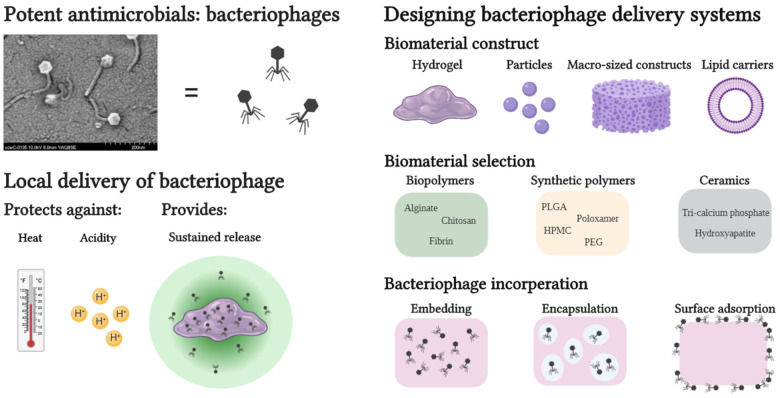

## Introduction to Bacteriophage Therapy

Bacteriophages (phages) are natural viruses which harbor specificity for a bacterial host. After the phage infects the bacterial host by inserting its genetic material through the bacterial cell wall and membrane, the host’s metabolism is rerouted to facilitate rapid phage replication. Toward the final stage of the bacteriophage replication cycle, phage-encoded endolysins disintegrate the bacterial cell wall from within, resulting in the release of the progeny phage. Phages are abundantly found in nature, including within the human body and contribute to homeostasis in the human microbiome.

Control of bacterial presence or numbers is necessary in many fields and disciplines, including food preservation (Founou et al., 2016), aquaculture (Culot et al., 2019), animal husbandry (Cuong et al., 2018), plant preservation (McManus et al., 2002), and medicine, and this is routinely achieved by classical antimicrobial chemotherapy. However, with the rise of antibiotic resistance, alternatives to antibiotic therapy are becoming ever more necessary; phage therapy offers one possible alternative (Ventola, 2015). However, the application of phages in medicine could be challenging to implement on a broad scale, as it would need to conform with strict national and international regulations (Furfaro et al., 2018).

Over a century ago, Felix d’Hérelle successfully treated the first patient using phages (Sulakvelidze and Kutter, 2004), although such early phage therapy reports were questioned due to a lack of control groups and reproducibility (Wittebole et al., 2014). Eventually, with the development of mass-produced antibiotics in the 1940’s, phages as an antimicrobial treatment were quickly forgotten in the western world. Phage research and treatments were, nevertheless, further developed in the former Soviet Union. Founded in 1923, the Eliava Institute in Georgia to this day continues to treat infected patients from all over the world using phages. In Wrocław, Poland, the Hirszfeld Institute has been founded in 1952 and remains the only specialized center for phage therapy in the European Union. In both Europe and the United States, recent clinical trials (as reviewed by Kutter et al., 2010 and Furfaro et al., 2018) have marked the first step toward applying phages to treat antibiotic resistant microbial infections. The growing global interest in phage therapy is evident by the increasing number of recent clinical trials using oral, intravenous or topical phage.

However, there are several limitations to the utilization of bacteriophages in medicine. Where antibiotics often show a broad-spectrum of applicability, bacteriophages often show lytic activity on a single species or strain of bacteria. In a clinical setting, this would necessitate identification of the bacterial strain prior to the start of phage treatment, which might not be possible in the case of acute infections (e.g., sepsis). This narrow host-range is beneficial in the sense that it has less impact on non-pathogenic commensal bacteria, while for effective treatments bacteriophage cocktails can be prepared, which increases the host range of phages against bacteria at a site of infection. Another limitation of bacteriophage treatment is the duration of its activity *in vivo*. Much like pharmaceuticals that have therapeutic half-lives, bacteriophages also experience a decay in their lytic activity over time. The clearance of intravenously injected bacteriophage is complete within 60 min (T7 phage) and the half-life of λ-phage was shown to be approximately 6 h (Prasuhn et al., 2008). Thus, to prevent *in vivo* bacteriophage decay, delivery strategies must be tailored to shield the phage from harmful environmental factors such as acidic/alkaline pH or serum inactivation. Sustained bacteriophage release from any biomaterial matrix locally implanted at the site of infection might prolong the effective treatment duration, resolving the necessity for repeated phage administration. This would be especially beneficial in the treatment of non-topical tissues such as bone where closure of the surgical site is highly desirable, and intravenous injection of phage is assumed to be ineffective.

In this review, we summarize the literature on the use of phage loaded biomaterials for enhanced stability and sustained release. While we maintain a primary focus on medical applications, we also include relevant literature on phage delivery in other fields (e.g., food industry), where appropriate. Finally, we offer what we consider promising advances in the field of infection control regarding combined phage treatments with antibiotics or the use of phage derived lysins.

## Advantages of Bacteriophage Loaded Biomaterials for Local Delivery

A major concern for the use of freely dispersed bacteriophage for medical treatments is their inactivation in physiological conditions. Firstly, exposure of bacteriophages to serum antibodies can result in immune clearance and reduction of phage infectivity (Prasuhn et al., 2008). Żaczek et al. (2016) has investigated the immune reaction of MS-1 phage on Anti-phage antibodies present in patients undergoing phage therapy. An increased phage inactivation rate was observed due to increased anti-phage antibodies present in patient serum, although clinical outcome was minimally influenced even for patients with high expression of anti-phage antibodies. However, in different clinical settings where initial phage concentrations are lower or where phages have difficulty reaching the target tissue, the phage inactivation by the immune system could be an additional adverse effect resulting in a failed treatment outcome.

Secondly, one of the harshest physiological conditions for bacteriophages is the acidic environment of the stomach, which limits efficient oral phage delivery to the gastrointestinal system. As was shown by Międzybrodzki et al., only a small fraction (<0.1%) of oral administered phage was observed in the intestines, even after neutralizing antacid administration. The rationale behind several reviewed studies is to overcome such problems by protecting bacteriophages against harmful environments through embedding in a biomaterial matrix (Kim et al., 2015; Moghtader et al., 2017; Abdelsattar et al., 2019; Otero et al., 2019; Vinner et al., 2019).

When repeated administration of freely dispersed phage is undesirable or impractical (e.g., for non-topical, deep-tissue/organ infections), implantation of phage-embedded biomaterials might lead to a sustained local release of the phage load. Without any means to propel itself, the embedded phage relies on diffusion to migrate from the biomaterial matrix in its search for a bacterial host (Barr et al., 2015). The *in vivo* pharmacokinetics of bacteriophages differ greatly from antibiotics in term of tissue uptake and diffusion due to the fact that most antibiotics are (small) molecules while phages are an agglomeration of proteins (Nilsson, 2019). This low mobility relative to antibiotics can be considered another benefit for local bacteriophage delivery, as phages would be released locally at the site of infection. Depending on the application, release kinetics of phages from a biomaterial matrix can be tuned by the biomaterial type and implant design (e.g., films, particles or hydrogels). Phage loading inside biomaterials (up to 10^11^ PFU/mL) often exceeds the phage concentration used in recently proposed phage treatment protocols for non-topical infections [10^7^ PFU/mL, with rinsing volumes ranging from 10 to 40 mL (Chan et al., 2018; Onsea et al., 2019)]. Due to the high phage load of biomaterials, a sustained release of loaded phage is achievable and may be beneficial in order to maintain a prolonged therapeutic activity of released phage. Alternatively, in a prophylactic scenario a burst release of phage can be considered beneficial. Huff et al. (2002) added *Escherichia coli* and SPR02 phage with two different multiplicities of infection (MOI), namely MOI = 1 (10^4^ CFU and 10^4^ PFU) and a MOI = 10,000 (10^4^ CFU and 10^8^ PFU), and observed an increased survival rate of chickens from 65% at MOI = 1 to 100% at MOI = 10,000 after oral administration of phage and bacteria. However, empirical evidence for a minimal effective local phage concentration for the treatment of local infections is not available and is most likely subject to phage-specific and patient-specific variability, making claims on “ideal” phage release kinetics challenging at the present time.

Another benefit of loading phage inside a biomaterial is that it allows storage of phage in off-the-shelf formulations. Lyophilization is a common approach that can yield phage formulations that are often stable under ambient storage conditions (Puapermpoonsiri et al., 2010). Successful lyophilization of phage requires the addition of cryoprotectants to prevent the disintegration of the phage by the freezing process (Puapermpoonsiri et al., 2010). The preparation of lyophilized phage loaded biomaterials where the biomaterial itself acted as a protectant against freezing stresses has been described (Puapermpoonsiri et al., 2009). By freeze-drying such a phage loaded construct, phage delivery systems with both long-term phage stability and storage capacity can be produced.

## Considerations in Manufacturing Bacteriophage Loaded Biomaterials

Due to the variety of bacteriophages described in the literature, and the widely recognized variability in their functionality under different conditions, it is difficult to make general statements on phage compatibility during incorporation with biomaterials. Thus, when incorporating bacteriophages on or in a biomaterial, the manufacturing procedure of the phage/biomaterial composite must be carefully considered and suited to the compatibility of the incorporated phage. Generally, three options are available for loading a biomaterial with bacteriophages: (1) embedding, (2) encapsulation, and (3) surface functionalization through phage adsorption or covalent binding. Which strategy is used is generally dictated by the type of biomaterial and its processability. For example, ceramics such as hydroxyapatite (HAP) or other calcium phosphates (CAP) require such high sintering temperatures (Meurice et al., 2012) that surface adsorption after sintering is the only viable method for direct phage loading for these materials. Other manufacturing processes such as organic solvent emulsions, electro-spinning/spraying or chemical modifications of biomaterials require conditions that could be harsh for the relatively fragile bacteriophages. The processing conditions that have been shown to be generally harmful to the viability of most phages were extremely acidic or alkaline environments (Jonczyk et al., 2011), high temperatures (Jonczyk et al., 2011), drying processes (Clark, 1962), ionic strength of solutions (Jensen and Kleppe, 1972), and mechanical stresses (Leung et al., 2019). Because varying observations have been made by different researchers when examining phage robustness, a freshly isolated bacteriophage should always be examined thoroughly in order to understand its limits before being subjected to processing with a biomaterial (Ackermann et al., 2004).

Strong alkaline or acidic environments have been recognized as detrimental for all types of bacteriophages, but a difference in tolerance has been reported between different phages. M13 and λ-phage, which are generally known as robust phages, can tolerate pH values between 3 and 11 for at least 20 min (Jepson and March, 2004; Branston et al., 2013), but phages with a lower tolerance to extreme pH values have also been reported (Kerby et al., 1949; Khawaja et al., 2016). Several groups have compared effects of extreme pH conditions and serum exposure of multiple bacteriophages and noticed significant differences in lytic activity between the tested phages (O’Flynn et al., 2006; Knezevic et al., 2011). It remains relatively unknown which factors or characteristics allow for a phage to possess a degree of acid resistance. The acidic pH-level of the stomach is a main concern for all orally applied phage formulations and can be addressed by embedding the phage in acid resistant polymer matrices (Zelasko et al., 2017).

The effect of high temperature on phage lytic activity has been studied extensively in order to understand the temperature range in which a phage can be processed and incorporated into a biomaterial. Seven species of bacteriophages were exposed to buffer at 60°C for 30 min, which led to titer reductions between 0.3 and 2.8log_10_ (Mocé-Llivina et al., 2003). Heat inactivation occurs very rapidly at higher temperatures with a complete inactivation of lactococcal phages in reconstituted skim milk held for 5 min at 90°C (Murphy et al., 2014). Zottola and Marth (1966) concluded that it took a *Streptococcus lactis* phage 200 min to be inactivated at 70°C, this only took 6 min at 77.8°C, signifying a rather precise temperature stability threshold.

Due to the heat-sensitivity of phages, steam-sterilization (generally at 121°C for 20 min) of phage-loaded biomaterials is not a viable option. Other sterilization techniques such as ethylene oxide (EtO) gas sterilization and gamma (γ) irradiation could potentially be applied to sterilize phage loaded biomaterials. Bienek et al. (2007) tested the effects of EtO gas sterilization and γ irradiation on lyophilized ΦX-174 bacteriophage powders. A 30-min sterilization protocol with EtO gas resulted in a 5.3log_10_ reduction. Following a standard γ-irradiation sterilization protocol, a 3.8log_10_ titer reduction was observed. The same study investigated the effects of EtO and γ sterilization when phage was inserted into cortical bone cavities which were sealed prior to the sterilization procedure, after which the phage was able to tolerate a 30-min EtO exposure. These results could imply that embedded or encapsulated bacteriophage can be sterilized by short EtO treatments. However, the quantity of research on sterilization of encapsulated bacteriophages is very limited. Other techniques for sterilization of phage loaded biomaterials such as Ultraviolet (UV) irradiation or chemical sterilization with hydrogen peroxide or nitrogen dioxide remain unexplored. Alternatively, any material used in bacteriophage embedding must be sterilized prior to the embedding procedure, which can present other logistical challenges. Due to a lack of phage loaded biomaterials in clinics, practical considerations such as device sterilization are often overlooked. This critical gap in the literature needs to be successfully addressed in order to facilitate rapid clinical translation of phage delivery biomaterials.

The presence of organic solvents and their volatility were also observed to be detrimental to phage activity (Salalha et al., 2006). Because of these practical limitations in applying organic solvents, water-soluble biopolymers such as sodium alginate are frequently used for bacteriophage encapsulation (Kim et al., 2015; Abdelsattar et al., 2019; Alves et al., 2019). Agarwal et al. (2018) could also prevent exposure of phages to organic solvents by adding them on poly(lactide-co-glycolic acid) (PLGA) porous microspheres, which allowed the phage to be directly absorbed.

Dry formulations of embedded phage are known to be beneficial for long term storage, as they allow stability at room temperature, making storage in refrigerated conditions unnecessary (Fortier and Moineau, 2009). Dry formulations of bacteriophages are also easily shipped and shared between labs, hospitals or potential industrial production plants without the need for wet or dry ice. The main techniques currently implemented for drying of bacteriophage formulations are ambient air drying (Ma et al., 2008), spray drying (Matinkhoo et al., 2011; Chang et al., 2017), lyophilization (Puapermpoonsiri et al., 2009) or spray freeze-drying (Leung et al., 2016). To protect bacteriophages from harmful drying effects during spray drying or lyophilization, (cryo-) protectants (i.e., skim milk, trehalose, sucrose) are added to the phage formulation. During lyophilization, cryoprotectants prevent damage to bacteriophages caused by the formation of ice crystals (Zhai et al., 2004). For spray drying, protectants shield the phage from thermal stresses and drying stresses (Matinkhoo et al., 2011). The necessity for (cryo-)protectants in drying processes of phage loaded materials has been demonstrated in control groups of several studies (Ma et al., 2008; Chang et al., 2017). Even upon addition of protectants, a 10-fold reduction in phage titer is typical after drying and long-term storage of phage (Ackermann et al., 2004) or phage loaded constructs (Ma et al., 2008). Ma et al. (2008) embedded *Staphylococcus aureus* phage K in alginate beads and proceeded to air-dry the beads that were soaked in solutions of protectants (i.e., skim milk, trehalose, sucrose) (Ma et al., 2012). High PFU titers were maintained with a 0.7log_10_ reduction observed in the most favorable condition (20% skim milk). While (cryo-)protectants can contribute to higher phage viability upon (freeze-)drying, reduced outcome of phage treatments when the applied phage products contain such (cryo-)protectants is a concern. Balogh et al. (2008) has reported that phage treatment in presence of skim milk failed to reduce bacterial spot disease severity on citrus fruits to the same extent as phage treatment without skim milk. Therefore, the need to remove (cryo-)protectants from phage products must be considered on a phage-to-phage basis, but also depending on the (cryo-)protectant used.

Besides environmental cues, some embedding processes can also be considered harmful to phages. Reduction of lytic activity by embedding phages in biomaterials is highly dependent upon the type of phage and biomaterial being used (Leung et al., 2019). For example, Leung et al. (2019) showed that long-tailed phages were more susceptible to structural damage after jet-nebulization compared to non-tailed phages. Before any large-scale phage incorporation endeavor can be made, a microscopic or microbiological analysis of the embedded phage should be made to assess the degree of structural damage and ensure phage/methodology compatibility. For an extensive and in-depth review on the effect of environmental factors on phage stability we recommend the reader to a review paper by Malik et al. (2017).

## Implementation of Biomaterials for Bacteriophage Delivery

### Organic Materials

#### Biopolymers

Biopolymers are polymeric macromolecules that can be produced by living organisms but can also be synthesized in industrial settings. Examples such as alginate, chitosan, and fibrin are frequently used for bacteriophage embedding studies, due to their mild processing conditions and high capacity for molecular tailoring (Bosio et al., 2011). See Table 1 for a literature summary on this topic.

**TABLE 1:** Overview of biopolymers used for bacteriophage embedding.

Envisioned application	Biopolymers and construct	Embedded phage	Relevant results				Reference
			*Study type*	*Phage release*	*Protective properties*	*Antimicrobial effects*	
Intestinal phage delivery	Millimeter sized beads with **sodium alginate and chitosan** core/shell structure. Alginate core viscosity was increased by supplementation with honey/gelatin.	*E. coli* phage ZSEC5	*In vitro*	Complete phage release in 5 h under SIF conditions.	Resisted acidic environments (pH = 2) for 60 min and 3-min exposure to 80°C, both with a 1log_10_ reduction of embedded PFU.	4.5–5log_10_ reduction of *E. coli* CFU after 10 h incubation.	Abdelsattar et al., 2019
Intestinal phage delivery	Microspheres of **sodium alginate and chitosan** core/shell structure.	*E. coli* phage O157:H7	*In vitro*	65% of embedded phage released after 6 h under SIF conditions.	Resisted acidic environments (pH = 2) for 60 min, with a 1.5log_10_ reduction of embedded PFU.	After 5 h incubation a 2.5log_10_ CFU reduction was observed compared to control cultures.	Kim et al., 2015
Intestinal phage delivery	Beads of **Sodium Alginate** with either a chitosan or a polyethyleneimine (PEI) coating	*E. coli* phage T4	*In vitro*	Release plateaus after 12 h under SIF conditions. Chitosan and PEI coatings delayed phage release but reduced cumulative release as well.	Polycationic coatings increased acid resistance of embedded phage. 4log_10_ reduction in Alginate beads vs. 1log_10_ reduction in Alginate-PEI beads.	*Not assessed*	Moghtader et al., 2017
Preservation of meat	**Sodium Alginate** thin films	*Pseudomonas fluorescens* phage ϕIBB-PF7A	*In vitro*	Complete release of phage in 3 h.	PFU count was reduced by 4.2log_10_ after storage of 12 weeks at 4°C.	*P. fluorescens* CFU was reduced by 2log_10_ compared to control cultures.	Alves et al., 2019
Phage delivery for non-topical infections	**Fibrin** glue (TISSEEL^®^, Baxter, United States)	*P. aeruginosa* phage PA01	*In vitro*	Phage release was followed for 11 days, in which daily release was between 10^9^ and 10^10^ PFU/day.	*Not assessed*	Significant reductions in OD_600_ indicate antimicrobial effects,	Rubalskii et al., 2019
Treatment of cystic fibrosis	Spray dried or lyophilized particles of a mixture of **trehalose, mannitol and leucine**	*P. aeruginosa* phage PEV2	*In vitro*	Phage delivery was in the order of 10^5^ PFU per dose from an inhaler device.	*Not assessed*	*Not assessed*	Leung et al., 2016
Intestinal phage delivery	**Sodium alginate and chitosan** beads (±800 μm diameter)	*Salmonellla* phage Felix O1	*In vitro*	Near complete phage release (1.6⋅10^7^ PFU) in 5 h under SIF conditions.	After 60 min in SGF (pH = 2.4) a 2.6log_10_ reduction in phage titer was observed. At pH = 2, no titer was observed after 30 min.	*Not assessed*	Ma et al., 2008
			***Study type***	***Phage release***	***Protective properties***	***Antimicrobial effects***	
Intestinal phage delivery	**Sodium Alginate/CaCO_3_**^–^ beads (±150 μm diameter)	*Salmonella* phages UAB_Phi20/Phi78/Phi87	*In vivo*	80–100% of all three phages were released after 40 min in SIF.	Titer loss after 60 min exposure to SGF approximately 2–3log_10_ for UAB_Phi78 and UAB_Phi87. No significant titer loss for UAB_Phi20.	Alginate/CaCO_3_^–^ beads showed 1.7log_10_ reduction of excremental *Salmonella.* Effect of free phage was initially stronger but showed no significant difference with control group a week after treatment ceased.	Colom et al., 2017
Prophylactic phage delivery in an osteointegrative hydrogel	**Sodium Alginate/nano-hydroxyapatite (nHAP)** hydrogel	*E. faecalis* phages vB_EfaS_LM99	*In vitro* and *ex vivo*	97% of phage released in 24 h. No difference in phage release observed for nHAP containing hydrogels.	Phage titers in the hydrogel are stable for 7 days	Approximate 2log_10_ reduction of CFU after coincubation of hydrogels with bacteria. On a chicken femur *ex vivo* model a 3log_10_ reduction of CFU was observed after 48 h.	Pennone et al., 2019
Phage delivery triggered by bacteria-secreted hyaluronidase	Composite films **of agarose** and **hyaluronan**	*S. aureus* phage ϕK	*In vitro*	Phage release of 10^6^ PFU/mL in 4 h of coincubation with hyaluronidase	*Not assessed*	*Not assessed*	Barros et al., 2020
Treatment of osteomyelitis after implant (i.e., screw) removal	**Alginate** hydrogel	CRISPR-Cas9 modified phage against *S. aureus*	*In vivo*	*Not assessed*	*Not assessed*	No significant difference between phage loaded alginate and empty alginate on CFU load of a bone defect. CFU reduction (0.55log_10_) was observed in the soft tissues surrounding the bone defect.	Cobb et al., 2019
**Clinical trial:** treatment of burn wound patients with *P. aeruginosa* monoinfections. (Phagoburn project)	**Alginate** wound dressing soaked in phage cocktail solution	Phage cocktail of 12 *P. aeruginosa* phages	*Clinical trial*	Phage release was assessed *in vitro*. Burst release of phages was observed. Data set was not published or referenced.	*Not assessed*	Phage loaded wound dressing performed less effectively as standard of care control (sulfadiazine silver cream). Less adverse side effects were observed in patients undergoing phage treatment.	Jault et al., 2019

*Abbreviations – CFU: Colony forming unit; nHAP: nano-hydroxyapatite; OD_600_: Optical density at a wavelength of 600 nm; PEI: Poly(ethylenimine); PFU: Plaque forming unit; SGF: Stimulated gastric fluid; SIF: Stimulated intestinal fluid.*

Alginate is a biocompatible polyanionic biopolymer and is widely used in food and pharmaceutical industries. On a molecular level, alginate consists of (1-4)-linked β-D-mannuronic acid (M) and α-L-guluronic acid (G) units. These M- and G-units make up the alginate polymer in homopolymer M or G sequences or in alternating MG blocks (Smidsrød and Skja°k-Br^-^— k, 1990). Alginate undergoes crosslinking (interaction of carboxylate groups on the G-units across two alginate chains) upon addition of divalent cations such as Mg^2+^, Sr^2+^, and Ca^2+^ (Lee and Mooney, 2012). Hence, alginates with a higher G content result in denser crosslinking and higher mechanical properties (Drury et al., 2004). This characteristic can be utilized for tailoring release kinetics when alginate materials are used for drug- or phage-delivery applications. Alginate does not undergo enzymatic degradation *in vivo* as mammals lack the specific enzymes necessary (Lee and Mooney, 2012). Diffusion of divalent ions out of the gel results in structural disintegration of the alginate hydrogel. Anionic properties of alginate make for an excellent material for oral bacteriophage delivery due to the non-swelling properties of alginate in acidic environments like the stomach, protecting embedded phage from the harsh acidity (Segale et al., 2016). The carboxylic acid groups of alginate are protonated in acidic environments, but at neutral pH levels (for example in the intestine), deprotonation of these carboxylic acids results in a great increase in swelling that allows for diffusion of drug or phages out of the alginate gels where it can be therapeutically active (Mumper et al., 1994). The size of the alginate construct seems to influence phage tolerance of acidic environments, as phage in millimeter-sized beads showed a 1log_10_ reduction (Abdelsattar et al., 2019) while phage embedded in microbeads with a diameter of ±150 μm expressed 2–3log_10_ reduction in phage titer (Colom et al., 2017).

Chitosan is another biopolymer that has attracted attention in the field of bacteriophage delivery. Implementing shells of chitosan around phage loaded matrices like alginate can slow down alginate swelling and thus phage or Ca^2+^ diffusion out of the particle, resulting in a prolonged stability of the alginate matrix and a longer therapeutic activity of the bacteriophage delivery system (Segale et al., 2016). From a chemical perspective, chitosan is a polysaccharide with repeating units of acetylated and non-acetylated forms of β-(1-4)-linked D-glucosamine. The solubility of chitosan is pH dependent as in acidic environments the protonation of its amine groups makes chitosan positively charged and water soluble. A multitude of studies have fabricated polyelectrolyte complexes of chitosan with anionic polymers such as alginate for drug delivery purposes (Ma et al., 2008; Kim et al., 2015; Abdelsattar et al., 2019). Cationic materials have been recognized as having antiviral properties, with a 1 mg/mL chitosan solution exerting a 2-log reduction of C2 and MS2 bacteriophages after coincubation for 1 min (Chatain-Ly et al., 2013). These antiviral properties argue for the use of chitosan mainly as a shell structure around constructs with embedded phage as opposed to a matrix for direct phage embedding.

Release kinetics of bacteriophages from polysaccharide matrices is relatively quick, with reports stating a complete release of embedded phage ranging from minutes to approximately 12 h in a neutral pH environment (Colom et al., 2017; Moghtader et al., 2017). Often, the envisioned application for many of these studies is to embed phages to provide protection against the acidic pH in the stomach, followed by a quick release of phage in the intestine. This is achieved by entanglement of the hydrogel polymers upon protonation of carboxylic groups in acidic environments and subsequent loosening of the hydrogel polymers in neutral pH environments (e.g., the gut) (Vinner et al., 2017). For intestinal targets, phage loaded alginate and alginate-chitosan materials are highly suitable, as a fast release of phage and disintegration of the alginate matrix progresses while the alginate passes the intestines (Rayment et al., 2009).

Recently, Rubalskii et al. (2019) used a commercially available fibrin glue (TISSEEL^®^) to embed bacteriophages and use this fibrin glue for *Pseudomonas aeruginosa* phage PA01 delivery to treat local infections. The fibrin matrix was completely dissolved after 11 days of incubation in saline solution but throughout this period, phages were consistently released at high titers of approximately 10^9^ PFU/mL. The prolonged phage release provided by this fibrin material may make it suitable to treat local infections (e.g., orthopedic or implant related infections). Further pre-clinical studies implementing TISSEEL^®^ adhesives for bacteriophage delivery would be of interest for rapid clinical translation of this established biomaterial.

In one of the few published studies describing *in vivo* investigations of phage loaded biomaterials, Colom et al. (2017) showed the embedding of three different *Salmonella* phages in alginate/CaCO_3_^–^ microbeads for intestinal delivery. The relatively small size of the alginate/CaCO_3_^–^ beads allowed for complete intestinal release of embedded phage in 40 min. Embedded phage or free phage was orally administered 1 day prior to oral inoculation with *Salmonella typhimurium* and continued daily for 8 days. When compared to control groups without treatment, a 1.7log_10_ CFU reduction of *S. typhimurium* was observed for the embedded phage treatment, even after administration had ceased for 1 week. In the case of non-embedded bacteriophages, *S. typhimurium* CFU increased to the same level as the non-treated control group within 24 h. The authors state that in all groups the *S. typhimurium* infection could not be completely prevented or eradicated due to high inoculation titers. Colom et al. (2017) states that an inoculation threshold that results in protection by phage released from alginate/CaCO_3_^–^ should therefore be established so that the prophylactic effectiveness of the phage delivery system can be determined.

A recent clinical trial under the name PhagoBurn consisted of testing an alginate based wound dressing loaded with a cocktail of 12 *P. aeruginosa* phages and was considered the first clinical trial of phage therapy following good manufacturing and clinical practice guidelines (Jault et al., 2019). In this clinical trial, commercially available alginate sheets were soaked in a phage solution to allow phage loading of the alginate construct. Subsequently, the alginate sheets were applied on *P. aeruginosa* infected burn wounds of randomized patients. This phage treatment was compared sulfadiazine silver cream treatment, a standard of care method consisting of a combination of sulfadiazine antibiotic and antimicrobial silver ions. The alginate wound dressings were able to reduce bacterial burden in the burn wounds, although no improved antimicrobial effects compared to the standard of care were observed. Due to these findings, the clinical trial was prematurely terminated, resulting in small number of recruited patients and low statistical power. However, the patients undergoing phage treatment did experienced less adverse events (e.g., septic shock, skin graft infection or impaired healing) compared the standard of care (23 vs. 54%, respectively), which is an interesting finding that exposes potential antimicrobial or immunological benefits for phage therapy. Even though the PhagoBurn clinical trial did not show superior antimicrobial efficacy compared to available therapies, it did set a precedent in terms of regulatory procedures for future clinical trials with optimized bacteriophage delivery.

#### Synthetic Polymers

A synthetic polymer is a collective name for many different polymer classes where the polymer molecule is artificially synthesized in laboratory or industrial settings. Synthetic polymers (e.g., polyesters, polyethers, polyurethanes, and polymethacrylates) make for attractive options as drug delivery systems due to the high control exerted over their physico-chemical properties. Several of these synthetic polymers have been implemented for delivery of bacteriophages. In contrast to the usually water-soluble biopolymers, organic solvents might be necessary for processing of synthetic polymers. Solvent choice must be compatible with encapsulated or embedded phage to provide effective delivery. Literature on this topic is summarized in Table 2, with an emphasis on the manufacturing procedures of the phage delivery systems. Co-formulations of biopolymers and synthetic polymers are also listed in Table 2.

**TABLE 2:** Overview of the use of synthetic polymers for bacteriophage embedding.

Envisioned application	Polymer and construct	Embedded phage	Relevant results				Reference
			*Study type*	*Manufacturing process and Protective properties*	*Phage release*	*Antimicrobial effects*	
Protection of phage against acidic gastric environment	Microcapsules of anionic **methacrylic acid** and **methyl methacrylate copolymer** (Eudragit^®^ S100) and **trehalose**.	*S. enterica* phage Felix O1	*In vitro*	Phages exposed to high temperatures during spray drying (100–180°C) with low titer loss. Copolymer: trehalose (2:1 ratio) showed high phage loading and acid resistance. Dry storage (RT, 3 months) caused 1log_10_ titer reduction.	Encapsulation of 10^9^ PFU/gram was achieved. Presence of trehalose prevented a 4log_10_ reduction observed for copolymer formulations.	*Not assessed*	Vinner et al., 2019
Food preservation by incorporation in packaging material	Hollow fibers mats of **poly (ethylene oxide) (PEO)** and **cellulose diacetate (CDA)** blends.	*E. coli* phage T4	*In vitro*	PEO and CDA were dissolved in 98:2 wt% chloroform/methanol. Fiber mats were fabricated by coaxial electrospinning. Fiber diameter range 1.35–2.47 μm, containing internalized phage.	Higher PEO M_W_ equals slower phage release kinetics. Increase of hydrophobic CDA slows fiber swelling and thus phage release kinetics.	*Not assessed*	Korehei and Kadla, 2014
Treatment of bacterial lung infections	W_1_/O/W_2_ Microparticles of **PLGA** and **Pluronic 92L**, fabricated by W/O/W emulsions	*S. aureus* phage and an isolated *P. aeruginosa* phage	*In vitro*	W_1_: 1% aqueous solution of Pluronic 92L, O: 5% PLGA solution in DCM, W_2_: 1% aqueous PVA solution. Both W_1_ and W_2_ contained phage. Emulsions were mixed by homogenizer. Microparticles were frozen in liquid N_2_ and lyophilized without cryoprotectants.	Encapsulated phage in W_1_ did not efficiently release. W_1_ and W_2_ combined phage release reached 15% and 25% of encapsulated *S. aureus* and *P. aeruginosa* phage, respectively.	Lytic activity of both *S. aureus* and *P. aeruginosa* phage prevented bacterial lawn formation after 1- or 3-days storage at room temperature. No PFU observed after 7 days dry storage at 4°C or RT.	Puapermpoonsiri et al., 2009
Treatment of root canal infections	**Poloxamer P407** hydrogel (30% w/v)	*E. faecalis* phages EFDG1 and EFLK1	*In vivo*	30% poloxamer P407 solution was made by directly dissolving the polymers in the phage cocktail dispersion (10^9^ PFU/mL).	Hydrogel gelation occurred in 2–3 min after incubation at 37°C. Released phage gradually decreased over 28 days (from 10^8^ PFU/mL to 10^4^ PFU/mL).	*In vitro* CFU reduction of 3–4log_10_ reduction against biofilm cultures. *In vivo* efficacy in a rat model was proven against a 4-week old root canal infection (2log_10_ CFU reduction) and was superior to a single 10^9^ PFU/mL administration of unembedded phage (1.5log_10_ CFU reduction).	Shlezinger et al., 2019
			***Study type***	***Manufacturing process and Protective properties***	***Phage release***	***Antimicrobial effects***	
Delivery of phage to the lungs of cystic fibrosis patients	Hollow microparticles of **poly (lactic-co-glycolic acid) (PLGA)**	Multiple *P. aeruginosa* phages	*In vivo*	Porous PLGA microparticles with phage loading after particle formation. Phage load of 2.6⋅10^6^ PFU/mg particle. Dry powder formulation by lyophilizing particles in lactose solutions.	Inhibitory zone of *P. aeruginosa* around phage loaded PLGA particles was microscopically observed, indicating phage release from the PLGA microparticle.	*In vitro* biofilm CFU reduction of 2.5log_10_. Mice inoculated with *P. aeruginosa* were treated by particle inhalation resulting in a 1.5log_10_ reduction of CFU compared to free phage administration. Survival rate upon treatment was 100% versus 13% for untreated control mice.	Agarwal et al., 2018
Prophylactic phage and antibiotic delivery on orthopedic implants (K-wires)	**Hydroxypropyl methylcellulose (HPMC)** coating on K-wires	MRSA phage MR-5	*In vitro*	2% and 4% HPMC solutions containing phage and/or linezolid were used for dip coating the K-wires.	From 2% HPMC coatings, release was observed for 48 h. For 4% HPMC coatings a release period of 96 h was reached.	Reduction of 3log_10_ of CFU adhered to the K-wire with HPMC-phage coating. Additive effect between linezolid and phage was evident (>4log_10_ reduction of adhered CFU) after 48 h.	Kaur et al., 2014
Prophylactic phage and antibiotic delivery on orthopedic implants (K-wires)	**Hydroxypropyl methylcellulose (HPMC)** coating on K-wires	MRSA phage MR-5	*In vivo*	4% HPMC solutions containing phage and/or linezolid were used for dip coating the K-wires.	*In vivo* release of phage was not tested. Outcome based on CFU reduction.	Bacterial load diminished fastest for antibiotics/phage combination. Phage, antibiotic and combined loaded HPMC coating resulted no CFU observed in the soft tissue surrounding the implant.	Kaur et al., 2016
Treatment of orthopedic infections	Hydrogel of **4-armed polyethylene glycol (PEG)** functionalized with crosslinking moieties	*P. aeruginosa* phages ΦPaer4/ ΦPaer14. *S. aureus* phage ΦK	*In vivo*	4 arms of the PEG macromers were functionalized with crosslinkers or tissue adhesive peptides.	Phage release dictated by enzymatic hydrogel degradation. In presence of collagenase, 50% phage release observed in 8 h.	*In vitro* anti-biofilm experiments resulting in a 1log_10_ reduction in CFU. *In vivo*, a 0.5log_10_ reduction in CFU was observed 7 days after phage/hydrogel treatment.	Bean et al., 2014

*Abbreviations - CDA: cellulose diacetate; CFU: Colony-forming unit; DCM: Dichloromethane; HPMC: Hydroxypropyl methylcellulose; MRSA: Multi-resistant *Staphylococcus aureus*; PEG: Poly(ethylene glycol); PEO: Poly(ethylene oxide); PFU: Plaque-forming unit; PLGA: Poly(lactic-co-glycolic acid); PVA: Poly(vinyl alcohol); RT: Room temperature.*

One of the most frequently applied synthetic polymers in biomedical technologies, including many drug delivery systems, is poly(ethylene glycol) (PEG) [also known as polyethylene oxide (PEO)]. Compatibility of PEG with bacteriophages is evident, as it is a frequently used method for concentrating bacteriophage via precipitation in PEG solutions (Thorner et al., 2000; Castro-Mejía et al., 2015; Antibody Design Labs, 2019). As a hydrophilic polymer, PEG constructs can swell or dissolve in aqueous environments which can in turn trigger release of embedded bacteriophages (Korehei and Kadla, 2014). Poloxamers are polymers whose structure and properties are very similar to PEG. Poloxamers consist of a hydrophobic polypropylene oxide (PPO) polymer with PEG chains on both chain-termini. Poloxamers show thermo-responsive behavior at elevated temperatures, and with the proper poloxamer composition (e.g., Poloxamer 407), undergo gelation at physiological temperatures. This is due to reduced solubility of the PPO segments upon temperature increase (Linse, 1993). Poloxamer 407 has been investigated as a delivery material for bacteriophages, showing gelation of a 30% Poloxamer 407 solution with phage dispersing in 2–3 min at physiological temperatures (Shlezinger et al., 2019). Poloxamer 407 hydrogels released bacteriophages over a 28-day period with daily released phage titers reducing from 10^8^ PFU at day 1 to 10^4^ PFU at day 28 (Shlezinger et al., 2019). With an *in vitro* phage delivery of approximately 1 month, such phage loaded hydrogel systems are a promising development for the treatment of infections where repeated phage administration is not viable or desirable (e.g., orthopedic infections).

The polyester poly(lactic-co-glycolic acid) (PLGA) is another synthetic polymer that has been used to embed or encapsulate bacteriophages (Puapermpoonsiri et al., 2009; Agarwal et al., 2018). Because of PLGA’s limited water uptake and swelling properties, bacteriophage release from PLGA materials is much slower compared to hydrogel systems. Agarwal and coworkers have fabricated porous PLGA microparticles for bacteriophage delivery to treat lung infection in cystic fibrosis patients (Agarwal et al., 2018). In order to have speedy and appropriate phage release kinetics from PLGA materials, the microparticles were additionally incubated in a buffered bacteriophage suspension to allow adsorption of phage into the microparticle pores and onto the surface. After inhalation of the PLGA microparticles, the local release of phage led to survival of mice that were administered a lethal inoculum of *P. aeruginosa*. Besides rapid release of phage from the microparticle, this approach has the additional advantage of preventing phage exposure to organic solvents that were used during PLGA microparticle fabrication.

Kaur et al. (2014, 2016) have implemented hydroxypropyl methylcellulose (HPMC) as a coating material for K-wires used in orthopedic surgery. HPMC is a synthetic polymer made from cellulose that is chemically modified by the addition of 2-hydroxypropyl ether groups on the polysaccharide chain. Combinations of phages and antibiotics were added to solutions of HPMC that were then used for repeated dip-coating of K-wires. *In vitro*, these coated K-wires loaded only with phages, released detectable quantities of phage for 4 days, resulting in a 3log_10_ CFU reduction of multi-resistant *S. aureus*. When the material containing both phage and antibiotics was tested, an additive antimicrobial effect was seen (Kaur et al., 2014). In a pre-clinical mouse model of infection in the knee joint, these coated implants with phage or antibiotic load resulted in complete microbial clearance 15 days after implantation of an inoculated K-wire, while for combined phage and antibiotic coating complete microbial clearance was achieved after 10 days (Kaur et al., 2016). It must be mentioned that after 21 days, the untreated control group also showed an absence of CFU, which might limit the validity of this pre-clinical model.

#### Liposomal Encapsulation

Liposomes are lipid bilayer spherical structures made from amphiphilic phospholipids. They present an interesting construct for intracellular bacteriophage delivery as the encapsulated bacteriophage is not embedded in a biomaterial matrix, but rather entrapped in a bilayer structure. Within a liposome, phages can be shielded from harmful environments while at the same time still being dispersed within the inner liposomal liquid. Intracellular delivery of bacteriophages could be achieved when the liposomal membrane fuses with eukaryotic cell membranes. It is currently debated if phages are able to diffuse through eukaryotic cell walls (Nieth et al., 2015b), making liposomes an attractive option for intracellular phage delivery. A very versatile selection of therapeutics can be loaded into liposomes as the aqueous core of liposomes is suitable for hydrophilic drugs or dispersed bacteriophages, while the bilayer shell can be loaded with hydrophobic drugs. Liposomes are compatible with many routes of administration, including parental, pulmonary and transdermal, while oral delivery requires specific additions (e.g., chitosan or PEG coatings) to the lipid bilayer to ensure the liposomes withstand acidic environments and bile salts of the gastrointestinal tract (Torchilin, 2005). Liposomes can be fabricated by several techniques including lipid bilayer (thin film) rehydration or agitation/sonication of phospholipid solutions. For a complete overview of liposome preparation strategies, we recommend the work by Akbarzadeh et al. (2013) which reviews liposome production methods and potential applications. Literature regarding liposomal phage carriers can be found in Table 3.

**TABLE 3:** Overview of the use of liposomes for bacteriophage encapsulation.

Envisioned application	Material and construct	Embedded phage	Relevant results				Reference
			*Study type*	*Manufacturing and encapsulation process*	*Phage release / Protective properties*	*Antimicrobial effects*	
Food processing industry	Niosomes, liposomes and transfersomes from commercially available phospholipid formulations (Pronanosome Lipo-N^TM^ and Pronanosome Nio-N^TM^)	Staphylococcal phage phiIPLA-RODI	*In vitro*	For liposomes and Niosomes. the commercial formulations were dissolved in 0.05M HEPES buffer. The solutions were agitated by manual shaking and homogenization. Transferosomes were fabricated with thin film hydration.	Niosomes and liposome showed a 2-log reduction in PFU after acid exposure. Niosomes protected the encapsulated phage against elevated temperatures of 60°C, again showing a 2-log reduction where other formulations expressed no active phage.	*Not Assessed*	Gonzalez-Menendez et al., 2018
Wound dressing	Uni-lamellar liposomes (diameter ± 200 nm) containing: ∙ Phosphatidylcholine ∙ Cholesterol ∙ Tween 80 ∙ Stearylamine (weight ratio: 7/3/1/0.5)	*S. aureus* phage MR-5 and MR-10	*In vivo*	Encapsulation efficiency of 87% for both phages.	Phage was quantifiable for 10 days at the site of the wound. Liposome entrapped phages initially showed higher local PFU count as free phage.	Encapsulated phage in liposomes performed similarly as the antibiotic control group, where free phage was unable to clear infection. Wound healing was more rapid for liposome treated group	Wroe et al., 2020
Uptake of encapsulated phage by intestinal cells	Liposomes containing: ∙ phospholipids ∙ cholesteryl ∙ polyethylene glycol 600 ∙ cholesterol ∙ **cholesteryl 3β-N-(dimethylaminoethyl) carbamate hydrochloride** (weight ratio: 1/0.1/0.2/0.7)	*S. enterica* phage UAB_Phi20	*In vivo*	10^11^ PFU/mL dispersed phage could be encapsulated though lipid layer hydration methods with an efficacy of 46%.	Upon oral administration in mice, phage labeled with infrared marker was detected in several organs, including the stomach, intestine, spleen, liver and muscle.	*Not assessed*	Otero et al., 2019
			***Study type***	***Manufacturing and encapsulation process***	***Phage release / Protective properties***	***Antimicrobial effects***	
Phage delivery for internal medicine	Liposomes containing: ∙ DSCP phospholipids ∙ Cholesterol	*S. aureus* phage K ATCC 1985-B1 and *E. coli* phage ATCC 11303-B3	*In vitro*	Liposomes were fabricated using microfluidic hydrodynamic flow focusing, with a phage load of ∼10^8^ PFU/mL	Encapsulation in liposomes offered limited protection against acidic environments, with a 3log_10_ reduction of phage.	*Not assessed*	Cinquerrui et al., 2018
Intestinal phage delivery	Liposomes (diameter ± 300 nm) containing: ∙ 1,2-dilauroyl-rac-glycero-3-phosphocholine (DOPC) ∙ Cholesteryl ∙ polyethylene glycol 600 sebacate ∙ cholesterol ∙ cholesteryl 3β-N-(dimethylaminoethyl) carbamate hydrochloride (molar ratio: 1/0.1/0.2/0.7)	*Salmonella* phages UAB_Phi20/Phi78/Phi87	*In vivo*	Liposomes formed by thin-film hydration method. Encapsulation efficiency between 47 and 49% for all three phages.	Liposomes provided limited protection against acidic environments (4–5log_10_ PFU titer reduction).	Presence of phage in the excrement of chickens is 90% for chickens where encapsulated phage was administered. For free phage, this was only 35%. After 15 days, a 2log^10^ reduction in Salmonella CFU was observed in the excrement of chickens treated with phage loaded liposomes.	Chhibber et al., 2018
Intracellular phage delivery	Liposomes (diameter ± 100 nm) ∙ Phosphatidyl choline ∙ Cholesterol ∙ Tween 80 ∙ Stearylamine (weight ratio: 9/1/2/0.5)	*K. pneumoniae* phage KPO1K2 (MTCC 5831)	*In vivo*	Thin film hydration at 40°C.	Liposomes provided full protection against phage neutralizing antibodies and serum. Liposome encapsulated phage was observed in the spleen of BALB/c mice for 14 days, while free phage was not quantified after 2 days.	95% intracellular bacteria were killed by liposome encapsulated phages, while only a 21% reduction was seen for free phage.	Singla et al., 2016
Intracellular phage delivery	Giant unilamellar Liposomes (diameter in μm range) ∙ DOPC ∙ 1,2-dioleoyl-sn-glycero-3-phospho-L-serine (weight ratio: 50/50)	*Mycobacterium smegmatis* phage TM4 and *E. coli* phage λeyfp	*In vitro*	Gel-assisted thin film hydration and inverse emulsion	Only intracellular phage presence was measured. Approximately 4-fold increase of intracellular phage was observed by fluorescent confocal microscopy.	*Not assessed*	Nieth et al., 2015a

*Abbreviations: CFU: Colony-forming units; HEPES: (4-(2-hydroxyethyl)-1-piperazineethanesulfonic acid); PFU: Plaque-forming units.*

Gonzalez-Menendez et al. (2018) investigated the viability of encapsulating phages with liposomes and other carriers called niosomes and transferosomes. Briefly, the membrane of niosomes consists of non-ionic surfactants (e.g., Tween 20, Triton X-100, etc.) and the membrane of transferosomes is supplemented with edge activators that allow vesicle deformability of the vesicle, allowing for high tissue permeability. All three vesicle types could be loaded efficiently with bacteriophages (10^7^ PFU/mL), with encapsulation efficiencies ranging between 96.6 and 99.8% (Gonzalez-Menendez et al., 2018). Niosomes were found to protect best against low pH and high temperature environments, as it is believed that the non-ionic surfactants in niosomes are more stable in these conditions. Intracellular delivery of bacteriophages with the liposomes, niosomes or transferosomes, or any other antimicrobial functionality was not described in this work.

Cinquerrui et al. (2018) reported on *S. aureus* K-phage and *E. coli* T3 phage encapsulation in phospholipid DSCP:cholesterol liposomes prepared by microfluidic hydrodynamic flow focusing. Increasing the cholesterol content of the lipid bilayer increased liposome size and broadened the liposomal size distribution. Significant differences were observed between the encapsulation efficiencies of K-phage and T3 phage with ∼10^5^ and ∼10^9^ PFU/mL, respectively. This discrepancy was attributed to a slow interaction between K-phage and the forming lipid bilayers, and to the phage size, with K-phage (and phage agglomerates) being too large to be effectively encapsulated. Interestingly, when testing encapsulation efficiency of phage in different initial titers, the highest titer (∼10^11^ PFU/mL) resulted in a lower quantity of phage as when a lower initial titer (∼10^10^ PFU/mL) was attempted. This indicates that a high phage titer can cause agglomeration and thus hinder efficient encapsulation (Cinquerrui et al., 2018). On a final note, Cinquerrui et al. (2018) also warns the reader about overestimation in published reports of phage encapsulation in liposomes. They suggest this is due to externally adsorbed phages being included in the phage titer quantification. These phages would not benefit from any of the protective effects of liposomal entrapment and thus might not reach the target infection, resulting in lower efficient phage dosages than expected.

With the consensus that bacteriophages are unable or inefficient in diffusing past cellular membranes, liposomes form an interesting carrier material for intracellular bacteriophage delivery. However, their efficacy in intracellular microbial clearance is not frequently reported, with only a hand-full of publications reporting successful intracellular phage delivery (Nieth et al., 2015a; Singla et al., 2016). Endeavors in this field could yield valuable information regarding the efficacy of phage loaded liposomes against intracellular infections.

### Inorganic Materials

Examples of inorganic materials include ceramics, metals and salts. Although not as frequently investigated as organic phage carriers, delivery of bacteriophages using inorganic materials has received some attention, especially in the field of orthopedic infections (Meurice et al., 2012; Hornez et al., 2013). Literature regarding inorganic phage carriers can be found in Table 4. Ceramics like tricalcium phosphate (TCP) or hydroxyapatite (HAP) provide a scaffold for osteoconduction and ultimately for repair of bone defects (Hornez et al., 2013) and the presence of bacteriophage could prevent the onset of infection at the defect site. Because of the high temperatures associated with ceramic sintering, phages must be adsorbed onto the construct after the fabrication process is complete. Meurice et al. (2012) prepared HAP and TCP pellets which were doped in a bacteriophage dispersion for 24 h under refrigerated conditions. An increase in porosity of the sintered pellets resulted in a delayed antimicrobial response, indicating a slower release of bacteriophage that were adsorbed on the inner porous structures of the pellets (Meurice et al., 2012). To show that the release of phages was not a mere initial burst release, the ceramic pellets were washed for 3 h prior to antimicrobial testing. The ceramics with the most porosity showed the highest inhibitory properties after such a pre-wash was performed, corroborating the results that showed delayed release of phage from porous ceramic pellets (Meurice et al., 2012).

**TABLE 4:** Overview of the use of non-polymeric materials for bacteriophage embedding.

Envisioned application	Material and construct	Embedded phage	Relevant results				Reference
			*Study type*	*Manufacturing process*	*Phage release / Protective properties*	*Antimicrobial effects*	
Intercellular delivery of bacteriophages	Biomimetic **HAP** nanocrystals	*S. rissen* phage SR φ1	*In vitro*	Phage and HA nanoparticles were mixed together, and phages adsorbed on the HA material. 10^7^ PFU/mg of HA nanoparticles were adsorbed after 24 h.	Phage adsorbed on HA nanocrystals show no reduction of lytic activity after 6 weeks of storage at 4°C. Adsorbed phage shows no inactivation due to acidic environment.	A 3-log reduction of *S. rissen* was observed when incubated with HA-SR φ1.	Fulgione et al., 2019
Delivery of phage within difficult to penetrate biofilms	**Chitosan** coated **iron oxide non-colloidal nanoparticles** for phage grafting	*E. coli* phage C3000 and *P. aeruginosa* phage PA01	*In vitro*	Phage is conjugated to the chitosan coated iron oxide nanoparticle though carbodiimide chemistry.	No phage release, particles possess contact killing though surface functionalized phages.	Particles were able to penetrate biofilms better under magnetic fields and reduce the quantity of biofilm with 90% (compared to 35% for free phage).	Li et al., 2017
Prophylactic use in orthopedic reconstruction surgery	Microporous pelleted **CaP** granules	*E. coli* phage λ vir	*In vitro*	Phage was loaded by allowing adsorption at 4°C for 24 h.	The pellets were washed for 3 h prior to testing, resulting in a 2-log reduction of phage load, indicating strong burst-like release properties. However, pellets washed for 6 days could still inhibit bacterial growth in a zone of inhibition experiment.	Optical density measurements show a reduction when phage loaded CaP materials were introduced to the culture. An increase of porosity of the sintered materials results in higher phage load and a lower optical density of the bacterial culture.	Meurice et al., 2012
Prophylactic use in orthopedic reconstruction surgery	Macro porous **CaCO_3_** and **HAP** scaffold.	*E. coli* phage λ vir	*In vitro*	Phage was loaded by allowing adsorption at 4°C for 24 h.	*Not assessed*	OD_600_ measurements indicate antimicrobial effects after 90 min of coincubation. Increased porosity	Colom et al., 2015

*Abbreviations – CaP: Calcium phosphate; HA: Hydroxyapatite; OD_600_: Optical density at a wavelength of 600 nm.*

Delivering phages intracellularly proves to be an additional challenge that must be overcome by designing appropriate delivery systems. Fulgione et al. (2019) used HAP nanoparticles (±300 nm diameter) with adsorbed bacteriophage to achieve intracellular phage delivery. Uptake of the HAP-phage complex in cancer cells was visualized by fluorescent microscopy of fluorescein isothiocyanate (FITC) labeled HAP. A 3log_10_ CFU reduction of intracellular *Salmonella* was observed for the HAP-phage complex while standalone phage caused no significant reduction of intracellular CFU (Fulgione et al., 2019). As part of the pathology of bone infection, *S. aureus* bacteria are also known to reside intracellularly where antimicrobial treatments might be less effective (Dusane et al., 2018). While *Salmonella* osteomyelitis is relatively rare, this intracellular phage delivery system could present a promising tool to target intracellular *S. aureus* bacteria.

In the case of HAP materials, the adsorption kinetics of SR φ1 phage appear to be driven by a difference in zeta potential (negative for most phages and slightly positive for biomimetic HAP), facilitating high phage adsorption in a time dependent manner (Fulgione et al., 2019). With adsorption of phage being a surface interaction, porous materials show a much higher loading capacity than denser materials (Meurice et al., 2012). Intuitively, one could expect surface adsorbed phage release to proceed (e.g., detach) in a speedy fashion. Even though phage loaded HAP and TCP materials did show a large burst release, TCP pellets that were washed for up to 6 days could still establish an inhibition zone in an agar diffusion assay, regardless of their porosity (Meurice et al., 2012).

Li et al. (2017) have attempted to improve biofilm penetration of bacteriophages by covalently binding *E. coli* and *P. aeruginosa* bacteriophage to chitosan coated magnetic iron oxide nanoparticles. Upon application of a magnetic field, the phage-functionalized nanoparticles were able to be pushed through a soft-agar bacterial lawn in the direction of the magnetic field. As an unconventionally diluted agarose solution was used (0.1%), it remains unclear if the *in vitro* model matches biofilms found *in vivo* and if nanoparticles would be able to penetrate such biofilms. Due to the small size of the delivery system (±100 nm), cells are expected to internalize this phage delivery system through endocytosis potentially allowing for efficient intracellular phage delivery. However, the use of these functionalized nanoparticles for intracellular phage delivery, and whether the phage was able to replicate was not investigated. While deviating from the bacteriophage delivery approach in which phage must be released from its carrier material, covalent bacteriophage grafting onto surfaces can be also be considered a strategy to prevent bacterial colonization of surfaces or medical implants (Hosseinidoust et al., 2011). However, such surfaces would need a homogeneous and dense grafting of a broad selection of phages in order to achieve the desired anti-fouling effect. In the author’s opinion, prophylactic phage release by one of the biomaterials reviewed in this work would be a more feasible strategy to prevent bacterial fouling of (medical) surfaces.

## Combined Delivery Strategies With Bacteriophages or Bacteriophage Derived Products

While many bacteriophage delivery systems have yet to undergo clinical trials, other strategies that implement combinations or elements of phage therapy are already being investigated. A few groups have experimented with delivery of phage derived lysins (Hathaway et al., 2017; Johnson et al., 2018) or combined delivery of phage and antibiotics (Kaur et al., 2016; Cobb et al., 2019). As phages require a bacterial host, the effects of a second antimicrobial on phage amplification kinetics must be considered. Investigations on the dual treatments of antibiotics and bacteriophages solutions have been investigated *in vitro* by several groups (Kumaran et al., 2018; Dickey and Perrot, 2019). Dickey and Perrot (2019) have investigated CFU reduction in biofilm experiments using nine different antibiotics at 2× MIC and 10× MIC concentration together with *S. aureus* phage isolated from a Eliava PYO cocktail. The phage-only treatment was able to reduce CFU in the biofilm by 1.3log_10_, and in this experimental setup could not eradicate the biofilm. For combined treatments they distinguished between simultaneous phage and antibiotic treatment or sequential treatment with phage followed by antibiotics. For simultaneous treatments, only enhanced bactericidal effects were seen for 2× MIC antibiotic concentrations, as adding phage to 10× MIC antibiotic treatment did not result in different outcomes and often severely reduced the presence of phage. For sequential phage and antibiotic treatments, results indicate that 7 out of 9 antibiotics at 2× MIC performed significantly better against biofilms. Without phage pre-treatment, only 1 antibiotic at 2× MIC showed antimicrobial effects against the *S. aureus* biofilms. These results show that the presence of phage conferred functionality of antibiotics at lower concentrations that would have been otherwise ineffective (Dickey and Perrot, 2019). A combined delivery system that first releases bacteriophages, followed by a secondary release of a moderate quantity of antibiotics seems to be a promising prospect in the search for efficient anti-biofilm therapies.

Bacteriophage-encoded endolysins degrade the bacterial peptidoglycan from within at the end of the viral replication cycle. Since the peptidoglycan is heavily responsible for maintaining cellular integrity (Silhavy et al., 2010), any breach in its structure ultimately leads to cell lysis and release of phage from its host, making endolysins key to the use of bacteriophages as bactericidal agents. To take advantage of their important function, endolysins, and other similar bacteriolytic enzymes such as bacteriocins, can be produced as recombinant proteins and applied to bacteria from the outside as an antimicrobial, where they exert their effect in an identical manner. As Gram-positive bacteria lack an outer cell membrane, they are particularly susceptible to the effects of endolysins and can be eradicated extremely rapidly (Schmelcher et al., 2012). Unlike bacteriophages or most other antimicrobials, endolysins do not distinguish whether their target bacterium is metabolically active, making them ideal for targeting cells that would otherwise evade metabolically dependent antimicrobial therapy (Briers et al., 2014). Endolysins are known to recognize and cleave highly conserved residues within the bacterial peptidoglycan, presumably making it extremely difficult for bacteria to evolve resistance to their function (Fischetti, 2005). Indeed, extensive efforts to generate endolysin resistant strains in the laboratory have proven unsuccessful, and resistance has never been observed (Loeffler et al., 2001; Schuch et al., 2002; Pastagia et al., 2011).

The combination of hydrogels with antibacterial enzymes has proven useful due to the stabilizing effects that polycationic polymers have when the two components are combined in a complex (Filatova et al., 2013). However, this can have negative effects on the enzymatic activity (Walsh et al., 2003), and has not been extensively explored. As with bacteriophages, hydrogels can be used for optimizing endolysin delivery to increase therapeutic efficacy, and a few studies have been performed using hydrogels to control their release. In one example, poly(*N*-Isopropylacrylamide) (PNIPAM) nanoparticles were successfully used *in vitro* to administer two bacteriolytic enzymes cystine- and histidine-dependent aminohydrolase/peptidase (CHAPK) and lysostaphin against *S. aureus* via thermally triggered release at 37°C (Hathaway et al., 2017). Also, lysostaphin has been successfully incorporated into a chitosan gel (Nithya et al., 2018). Encapsulation within such a hydrogel, while it does not necessarily form a complex, is thought to not only improve delivery, but to also enhance enzyme stability. This was proven in a recent prominent study which successfully used an injectable PEG-based hydrogel to deliver lysostaphin as a prophylactic against *S. aureus* orthopedic implant infection (Johnson et al., 2018). As a preventative measure, this method performed better than standard antibiotic therapy, and was improved upon in a later study which additionally incorporated bone morphogenetic protein-2 (BMP-2) into the same PEG-based hydrogel to promote healing of segmental bone defects (Kumaran et al., 2018). It remains to be seen how the local delivery of bacteriolytic enzymes can be used to treat established infections, but as recent studies have demonstrated their effectiveness in eradicating biofilms (Gutierrez et al., 2014; Olsen et al., 2018; Johnson et al., 2019), their outlook seems promising.

## Limitations and Critical Gaps in the Reviewed Literature

The antimicrobial potential of bacteriophages has been known for over a century, but large-scale clinical application of phages is still not implemented in most parts of the world. This originates from the fact that there are some critical gaps in our understanding of bacteriophage treatment and its effects on the patient and the patient’s immune system, which complicates any endeavors to overcome key disadvantages through phage delivery with biomaterials. It is important to address that ideal bacteriophage concentrations and duration of phage treatment are not scientifically established. Without this knowledge it is challenging to design optimized phage delivering biomaterials with ideal phage release kinetics. More animal studies or even clinical trials studying these treatment parameters would give valuable insights that can set aims in terms of release kinetics for phage delivery studies with biomaterials.

Medical use of bacteriophages is not limited to monotherapies, and *in vitro* testing of combined therapy with antibiotic or phage-encoded endolysin delivery is a relatively recent development which requires further investigation. Open questions remaining for further investigation include assessing the immune response to administered bacteriophage loaded biomaterials and the potential adverse effects of these biomaterials on patients with impaired immune status. Combinational therapies with anti-inflammatory agents would be another research focus which could potentially yield useful insights in suppressing adverse immune responses at the site of infection.

Most reviewed studies assessed the release and functionality of biomaterials loaded with a limited number of different phages. Understandably from an academic standpoint this can be considered a good start when designing new biomaterials for phage delivery, but for clinical application, a phage loaded biomaterial should be able to release a wide variety of phages or even a cocktail of phages. Efficient delivery of a phage cocktail becomes even more important during treatment of acute infections or prophylaxis when the specific microbes are not previously identified. The wide range of architecture (e.g., tailed vs. non-tailed) and size of bacteriophages makes variations in phage release kinetics from a biomaterial a possible concern. A big step toward clinical translation is made if biomaterials that show potential in phage delivery are further characterized for delivery of phage cocktails.

A practical limitation of bacteriophage loaded biomaterials is finding compatible sterilization techniques. The thermosensitive phages cannot undergo high temperature or steam sterilization and the few studies that focused on sterilization of phage loaded biomaterials showed that other common approaches such as EtO gas and γ-irradiation also reduced phage viability significantly (Bienek et al., 2007). Alternatively, the phage incorporation with biomaterials could occur under sterile conditions, but this invites other logistical difficulties and might go against clinical practices of re-sterilizing equipment prior to surgery. Other, more uncommon sterilization techniques such as UV and nitrogen dioxide remain to be investigated and should be pursued in order to find appropriate strategies for sterilization of phage loaded biomaterials.

## Conclusion

In conclusion, bacteriophage treatment represents a promising alternative to eradicate multidrug resistant bacteria. However, there are factors that limit their large-scale medical applicability, such as loss of activity in physiological conditions, which means they normally need to be repeatedly administered. As summarized in Tables 1–4, large log_10_ reductions of bacterial burden were observed when incorporation of phage in a biomaterial matrix could establish a highly concentrated local release of phages in proximity to the bacteria. Treatment of different infections requires the phage to be incorporated in a variety of biomaterial constructs. Phage delivery to the gut requires an acid resistant material, like alginate, for efficient phage deposition, while phage adsorbed to nanomaterial could enable the phage to infect intracellular bacteria. Embedding, encapsulating or adsorbing bacteriophages in or onto carrier materials can potentially expose the phage to adverse conditions, to which the response can be very phage specific. Thorough compatibility testing and characterization is therefore required before any phage be loaded into a biomaterial. With the wide range of available biomaterials, and a vast number of differently behaving bacteriophages, much experimental work must be performed to optimize the bacteriophage delivery and patient outcome. Nevertheless, against a background of bacterial antibiotic resistance, new and exciting phage delivery systems are expected to make their appearance in clinical trials and hopefully in the clinics in order to alleviate infection burden in global healthcare.

## Author Contributions

All authors have contributed significantly to the establishment of this manuscript, proofreading, and correcting the manuscript. SR, ES, and RZ have conducted literature search and have contributed to writing the manuscript. DG, RR, DE, and TM have conceptualized the need for a literature review on this emerging topic.

## Conflict of Interest

The authors declare that the research was conducted in the absence of any commercial or financial relationships that could be construed as a potential conflict of interest.

## References

[B1] Abdelsattar A. S. Abdelrahman F. Dawoud A. Connerton I. F. El-Shibiny A. (2019). Encapsulation of E. coli phage ZCEC5 in chitosan–alginate beads as a delivery system in phage therapy. *AMB Express* 9:87. 10.1186/s13568-019-0810-9 PMC6579803 31209685

[B2] Ackermann H.-W. Tremblay D. Moineau S. (2004). Long-term bacteriophage preservation. *WFCC Newslett.* 38 35–40.

[B3] Agarwal R. Johnson C. T. Imhoff B. R. Donlan R. M. McCarty N. A. García A. J. (2018). Inhaled bacteriophage-loaded polymeric microparticles ameliorate acute lung infections. *Nat. Biomed. Eng.* 2 841–849. 10.1038/s41551-018-0263-5 30854250 PMC6408147

[B4] Akbarzadeh A. Rezaei-Sadabady R. Davaran S. Joo S. W. Zarghami N. Hanifehpour Y. (2013). Liposome: classification, preparation, and applications. *Nanoscale Res. Lett.* 8:102. 10.1186/1556-276X-8-102 PMC3599573 23432972

[B5] Alves D. Marques A. Milho C. Costa M. J. Pastrana L. M. Cerqueira M. A. (2019). Bacteriophage ϕIBB-PF7A loaded on sodium alginate-based films to prevent microbial meat spoilage. *Int. J. Food Microbiol.* 291 121–127. 10.1016/j.ijfoodmicro.2018.11.026 30496941

[B6] Antibody Design Labs (2019). *Small-Scale Preparation of Filamentous Bacteriophage by PEG Precipitation 2015.* Available online at: http://www. abdesignlabs.com/technical-resources/bacteriophage-preparation/ (accessed November 27, 2019).

[B7] Balogh B. Canteros B. I. Stall R. E. Jones J. B. (2008). Control of citrus canker and citrus bacterial spot with bacteriophages. *Plant Dis.* 92 1048–1052. 10.1094/pdis-92-7-1048 30769518

[B8] Barr J. J. Auro R. Sam-Soon N. Kassegne S. Peters G. Bonilla N. (2015). Subdiffusive motion of bacteriophage in mucosal surfaces increases the frequency of bacterial encounters. *Proc. Natl. Acad. Sci. U.S.A.* 112 13675–13680. 10.1073/pnas.1508355112 26483471 PMC4640763

[B9] Barros J. A. R. Melo, LDRd, Silva, RARd, Ferraz M. P. (2020). Encapsulated bacteriophages in alginate-nanohydroxyapatite hydrogel as a novel delivery system to prevent orthopedic implant-associated infections. *Nanomedicine* 24:102145. 10.1016/j.nano.2019.102145 31857183

[B10] Bean J. E. Alves D. R. Laabei M. Esteban P. P. Thet N. T. Enright M. C. (2014). Triggered release of bacteriophage K from agarose/hyaluronan hydrogel matrixes by staphylococcus aureus virulence factors. *Chem. Mater.* 26 7201–7208. 10.1021/cm503974g

[B11] Bienek C. MacKay L. Scott G. Jones A. Lomas R. Kearney J. N. (2007). Development of a bacteriophage model system to investigate virus inactivation methods used in the treatment of bone allografts. *Cell Tissue Bank.* 8 115–124. 10.1007/s10561-006-9018-8 17061148

[B12] Bosio V. Islan G. Martinez Y. Castro G. (2011). “Control release applications in food technology,” in *Advances in Bioprocesses in Food Industry*, eds Soccol C. R. Pandey A. Soccol V. T. Laroche C. (New Delhi: Asiatech), 1–13.

[B13] Branston S. D. Stanley E. C. Ward J. M. Keshavarz-Moore E. (2013). Determination of the survival of bacteriophage M13 from chemical and physical challenges to assist in its sustainable bioprocessing. *Biotechnol. Bioprocess Eng.* 18 560–566. 10.1007/s12257-012-0776-9

[B14] Briers Y. Walmagh M. Grymonprez B. Biebl M. Pirnay J. P. Defraine V. (2014). Art-175 is a highly efficient antibacterial against multidrug-resistant strains and persisters of *Pseudomonas aeruginosa*. *Antimicrob. Agents Chemother.* 58 3774–3784. 10.1128/aac.02668-14 24752267 PMC4068523

[B15] Castro-Mejía J. L. Muhammed M. K. Kot W. Neve H. Franz C. M. A. P. Hansen L. H. (2015). Optimizing protocols for extraction of bacteriophages prior to metagenomic analyses of phage communities in the human gut. *Microbiome* 3:64. 10.1186/s40168-015-0131-4 PMC4650499 26577924

[B16] Chan B. K. Turner P. E. Kim S. Mojibian H. R. Elefteriades J. A. Narayan D. (2018). Phage treatment of an aortic graft infected with *Pseudomonas aeruginosa*. *Evol. Med. Public Health* 2018 60–66. 10.1093/emph/eoy005 29588855 PMC5842392

[B17] Chang R. Y. Wong J. Mathai A. Morales S. Kutter E. Britton W. (2017). Production of highly stable spray dried phage formulations for treatment of *Pseudomonas aeruginosa* lung infection. *Eur. J. Pharmaceut. Biopharmaceut.* 121 1–13. 10.1016/j.ejpb.2017.09.002 28890220 PMC5650506

[B18] Chatain-Ly M. H. Moussaoui S. Rigobello V. Demarigny Y. Vera A. (2013). Antiviral effect of cationic compounds on bacteriophages. *Front. Microbiol.* 4:46. 10.3389/fmicb.2013.00046 23487495 PMC3594988

[B19] Chhibber S. Kaur J. Kaur S. (2018). Liposome entrapment of bacteriophages improves wound healing in a diabetic mouse MRSA infection. *Front. Microbiol.* 9:561. 10.3389/fmicb.2018.00561 29651276 PMC5884882

[B20] Cinquerrui S. Mancuso F. Vladisavljević G. T. Bakker S. E. Malik D. J. (2018). Nanoencapsulation of bacteriophages in liposomes prepared using microfluidic hydrodynamic flow focusing. *Front. Microbiol.* 9:2172. 10.3389/fmicb.2018.02172 30258426 PMC6144953

[B21] Clark W. A. (1962). Comparison of several methods for preserving bacteriophages. *Appl. Microbiol.* 10 466–471. 10.1128/aem.10.5.466-471.1962 14021544 PMC1057894

[B22] Cobb L. H. Park J. Swanson E. A. Beard M. C. McCabe E. M. Rourke A. S. (2019). CRISPR-Cas9 modified bacteriophage for treatment of Staphylococcus aureus induced osteomyelitis and soft tissue infection. *PLoS One* 14:e0220421. 10.1371/journal.pone.0220421 31756187 PMC6874295

[B23] Colom J. Cano-Sarabia M. Otero J. Aríñez-Soriano J. Cortés P. Maspoch D. (2017). Microencapsulation with alginate/CaCO3: a strategy for improved phage therapy. *Sci. Rep.* 7:41441. 10.1038/srep41441 PMC5264180 28120922

[B24] Colom J. Cano-Sarabia M. Otero J. Cortés P. Maspoch D. Llagostera M. (2015). Liposome-encapsulated bacteriophages for enhanced oral phage therapy against *Salmonella* spp. *Appl. Environ. Microbiol.* 81 4841–4849. 10.1128/aem.00812-15 25956778 PMC4551199

[B25] Culot A. Grosset N. Gautier M. (2019). Overcoming the challenges of phage therapy for industrial aquaculture: a review. *Aquaculture* 513:734423 10.1016/j.aquaculture.2019.734423

[B26] Cuong N. V. Padungtod P. Thwaites G. Carrique-Mas J. J. (2018). Antimicrobial usage in animal production: a review of the literature with a focus on low- and middle-income countries. *Antibiotics (Basel)* 7:75. 10.3390/antibiotics7030075 30111750 PMC6164101

[B27] Dickey J. Perrot V. (2019). Adjunct phage treatment enhances the effectiveness of low antibiotic concentration against *Staphylococcus aureus* biofilms in vitro. *PLoS One* 14:e0209390. 10.1371/journal.pone.0209390 30650088 PMC6334939

[B28] Drury J. L. Dennis R. G. Mooney D. J. (2004). The tensile properties of alginate hydrogels. *Biomaterials* 25 3187–3199. 10.1016/j.biomaterials.2003.10.002 14980414

[B29] Dusane D. H. Kyrouac D. Petersen I. Bushrow L. Calhoun J. H. Granger J. F. (2018). Targeting intracellular Staphylococcus aureus to lower recurrence of orthopaedic infection. *J. Orthopaed. Res.* 36 1086–1092. 10.1002/jor.23723 28885721

[B30] Filatova L. Y. Donovan D. M. Becker S. C. Lebedev D. N. Priyma A. D. Koudriachova H. V. (2013). Physicochemical characterization of the staphylolytic LysK enzyme in complexes with polycationic polymers as a potent antimicrobial. *Biochimie* 95 1689–1696. 10.1016/j.biochi.2013.04.013 23665361

[B31] Fischetti V. A. (2005). Bacteriophage lytic enzymes: novel anti-infectives. *Trends Microbiol.* 13 491–496. 10.1016/j.tim.2005.08.007 16125935

[B32] Fortier L.-C. Moineau S. (2009). Phage production and maintenance of stocks, including expected stock lifetimes. *Methods Mol. Biol.* 501 203–219. 10.1007/978-1-60327-164-6_19 19066823

[B33] Founou L. L. Founou R. C. Essack S. Y. (2016). Antibiotic resistance in the food chain: a developing country-perspective. *Front. Microbiol.* 7:1881. 10.3389/fmicb.2016.01881 27933044 PMC5120092

[B34] Fulgione A. Ianniello F. Papaianni M. Contaldi F. Sgamma T. Giannini C. (2019). Biomimetic hydroxyapatite nanocrystals are an active carrier for *salmonella* bacteriophages. *Int. J. Nanomed.* 14 2219–2232. 10.2147/ijn.s190188 30992664 PMC6445186

[B35] Furfaro L. L. Payne M. S. Chang B. J. (2018). Bacteriophage therapy: clinical trials and regulatory hurdles. *Front. Cell Infect. Microbiol.* 8:376. 10.3389/fcimb.2018.00376 30406049 PMC6205996

[B36] Gonzalez-Menendez E. Fernandez L. Gutierrez D. Pando D. Martinez B. Rodriguez A. (2018). Strategies to encapsulate the *Staphylococcus aureus* bacteriophage phiIPLA-RODI. *Viruses* 10:495. 10.3390/v10090495 30217072 PMC6163856

[B37] Gutierrez D. Ruas-Madiedo P. Martinez B. Rodriguez A. Garcia P. (2014). Effective removal of *Staphylococcal* biofilms by the endolysin LysH5. *PLoS One* 9:e107307. 10.1371/journal.pone.0107307 25203125 PMC4159335

[B38] Hathaway H. Ajuebor J. Stephens L. Coffey A. Potter U. Sutton J. M. (2017). Thermally triggered release of the bacteriophage endolysin CHAPK and the bacteriocin lysostaphin for the control of methicillin resistant *Staphylococcus aureus* (MRSA). *J. Control. Release* 245 108–115. 10.1016/j.jconrel.2016.11.030 27908758 PMC5234552

[B39] Hornez J. C. Bouchart F. Meurice E. Descamps M. Leriche A. (2013). “Synthesis and fabrication of porous calcium phosphate ceramics for antibacterial bone substitutes,” in *Proceedings of the MATEC Web of Conferences*, Vol. 7 Maubeuge.

[B40] Hosseinidoust Z. Van de Ven T. G. Tufenkji N. (2011). Bacterial capture efficiency and antimicrobial activity of phage-functionalized model surfaces. *Langmuir* 27 5472–5480. 10.1021/la200102z 21452812

[B41] Huff W. E. Huff G. R. Rath N. C. Balog J. M. Xie H. Moore P. A. Jr. (2002). Prevention of *Escherichia coli* respiratory infection in broiler chickens with bacteriophage (SPR02). *Poultry Sci.* 81 437–441. 10.1093/ps/81.4.437 11998827

[B42] Jault P. Leclerc T. Jennes S. Pirnay J. P. Que Y.-A. Resch G. (2019). Efficacy and tolerability of a cocktail of bacteriophages to treat burn wounds infected by *Pseudomonas aeruginosa* (PhagoBurn): a randomised, controlled, double-blind phase 1/2 trial. *Lancet Infect. Dis.* 19 35–45. 10.1016/s1473-3099(18)30482-1 30292481

[B43] Jensen H. B. Kleppe K. (1972). Effect of ionic strength, pH, amines and divalent cations on the lytic activity of T4 lysozyme. *Eur. J. Biochem.* 28 116–122. 10.1111/j.1432-1033.1972.tb01891.x 4559097

[B44] Jepson C. D. March J. B. (2004). Bacteriophage lambda is a highly stable DNA vaccine delivery vehicle. *Vaccine* 22 2413–2419. 10.1016/j.vaccine.2003.11.065 15193403

[B45] Johnson C. T. Sok M. C. P. Martin K. E. Kalelkar P. P. Caplin J. D. Botchwey E. A. (2019). Lysostaphin and BMP-2 co-delivery reduces &lt;em&gt;S. aureus&lt;/em&gt; infection and regenerates critical-sized segmental bone defects. *Sci. Adv.* 5:eaaw1228. 10.1126/sciadv.aaw1228 31114804 PMC6524983

[B46] Johnson C. T. Wroe J. A. Agarwal R. Martin K. E. Guldberg R. E. Donlan R. M. (2018). Hydrogel delivery of lysostaphin eliminates orthopedic implant infection by *Staphylococcus aureus* and supports fracture healing. *Proc. Natl. Acad. Sci. U.S.A.* 115 E4960–E4969. 29760099 10.1073/pnas.1801013115 PMC5984524

[B47] Jonczyk E. Klak M. Miedzybrodzki R. Gorski A. (2011). The influence of external factors on bacteriophages–review. *Folia Microbiol.* 56 191–200. 10.1007/s12223-011-0039-8 21625877 PMC3131515

[B48] Kaur S. Harjai K. Chhibber S. (2014). Bacteriophage mediated killing of *Staphylococcus aureus* in vitro on orthopaedic K wires in presence of linezolid prevents implant colonization. *PLoS One* 9:e90411. 10.1371/journal.pone.0090411 24594764 PMC3940871

[B49] Kaur S. Harjai K. Chhibber S. (2016). In Vivo assessment of phage and linezolid based implant coatings for treatment of methicillin resistant *S. aureus* (MRSA) mediated orthopaedic device related infections. *PLoS One* 11:e0157626. 10.1371/journal.pone.0157626 27333300 PMC4917197

[B50] Kerby G. P. Gowdy R. A. Dillon E. S. Dillon M. L. Csâky T. Z. Sharp D. G. (1949). Purification, pH Stability and sedimentation properties of the T&lt;sub&gt;7&lt;/sub&gt; bacteriophage of *Escherichia Coli*. *J. Immunol.* 63:93. 18139410

[B51] Khawaja K. Abbas Z. Rehman S. (2016). Isolation and characterization of lytic phagesTSE1-3 against *Enterobacter cloacae*. *Open Life Sci.* 11 287–292.

[B52] Kim S. Jo A. Ahn J. (2015). Application of chitosan–alginate microspheres for the sustained release of bacteriophage in simulated gastrointestinal conditions. *Int. J. Food Sci. Technol.* 50 913–918. 10.1111/ijfs.12736

[B53] Knezevic P. Obreht D. Curcin S. Petrusic M. Aleksic V. Kostanjsek R. (2011). Phages of *Pseudomonas aeruginosa*: response to environmental factors and in vitro ability to inhibit bacterial growth and biofilm formation. *J. Appl. Microbiol.* 111 245–254. 10.1111/j.1365-2672.2011.05043.x 21554503

[B54] Korehei R. Kadla J. F. (2014). Encapsulation of T4 bacteriophage in electrospun poly(ethylene oxide)/cellulose diacetate fibers. *Carbohydrate Polymers* 100 150–157. 10.1016/j.carbpol.2013.03.079 24188849

[B55] Kumaran D. Taha M. Yi Q. Ramirez-Arcos S. Diallo J.-S. Carli A. (2018). Does treatment order matter? investigating the ability of bacteriophage to augment antibiotic activity against staphylococcus aureus biofilms. *Front. Microbiol.* 9:127. 10.3389/fmicb.2018.00127 29459853 PMC5807357

[B56] Kutter E. De Vos D. Gvasalia G. Alavidze Z. Gogokhia L. Kuhl S. (2010). Phage therapy in clinical practice: treatment of human infections. *Curr. Pharmaceut. Biotechnol.* 11 69–86. 10.2174/138920110790725401 20214609

[B57] Lee K. Y. Mooney D. J. (2012). Alginate: properties and biomedical applications. *Prog. Polym Sci.* 37 106–126. 10.1016/j.progpolymsci.2011.06.003 22125349 PMC3223967

[B58] Leung S. S. Y. Carrigy N. B. Vehring R. Finlay W. H. Morales S. Carter E. A. (2019). Jet nebulization of bacteriophages with different tail morphologies–Structural effects. *Int. J. Pharmaceut.* 554 322–326. 10.1016/j.ijpharm.2018.11.026 30445174

[B59] Leung S. S. Y. Parumasivam T. Gao F. G. Carrigy N. B. Vehring R. Finlay W. H. (2016). Production of inhalation phage powders using spray freeze drying and spray drying techniques for treatment of respiratory infections. *Pharm Res. Dordr.* 33 1486–1496. 10.1007/s11095-016-1892-6 26928668 PMC5083036

[B60] Li L. L. Yu P. Wang X. Yu S. S. Mathieu J. Yu H. Q. (2017). Enhanced biofilm penetration for microbial control by polyvalent phages conjugated with magnetic colloidal nanoparticle clusters (CNCs). *Environ. Sci.* 4 1817–1826. 10.1039/c7en00414a

[B61] Linse P. (1993). Micellization of poly(ethylene oxide)-poly(propylene oxide) block copolymers in aqueous solution. *Macromolecules* 26 4437–4449. 10.1021/ma00069a007

[B62] Loeffler J. M. Nelson D. Fischetti V. A. (2001). Rapid killing of Streptococcus pneumoniae with a bacteriophage cell wall hydrolase. *Science (New York N. Y.)* 294 2170–2172. 10.1126/science.1066869 11739958

[B63] Ma Y. Pacan J. C. Wang Q. Sabour P. M. Huang X. Xu Y. (2012). Enhanced alginate microspheres as means of oral delivery of bacteriophage for reducing *Staphylococcus aureus* intestinal carriage. *Food Hydrocolloids* 26 434–440. 10.1016/j.foodhyd.2010.11.017

[B64] Ma Y. Pacan J. C. Wang Q. Xu Y. Huang X. Korenevsky A. (2008). Microencapsulation of bacteriophage felix O1 into chitosan-alginate microspheres for oral delivery. *Appl. Environ. Microbiol.* 74 4799–4805. 10.1128/aem.00246-08 18515488 PMC2519356

[B65] Malik D. J. Sokolov I. J. Vinner G. K. Mancuso F. Cinquerrui S. Vladisavljevic G. T. (2017). Formulation, stabilisation and encapsulation of bacteriophage for phage therapy. *Adv. Coll. Interface Sci.* 249 100–133. 10.1016/j.cis.2017.05.014 28688779

[B66] Matinkhoo S. Lynch K. H. Dennis J. J. Finlay W. H. Vehring R. (2011). Spray-dried respirable powders containing bacteriophages for the treatment of pulmonary infections. *J. Pharmaceut. Sci.* 100 5197–5205. 10.1002/jps.22715 22020816

[B67] McManus P. S. Stockwell V. O. Sundin G. W. Jones A. L. (2002). Antibiotic use in plant agriculture. *Ann. Rev. Phytopathol.* 40 443–465. 12147767 10.1146/annurev.phyto.40.120301.093927

[B68] Meurice E. Rguiti E. Brutel A. Hornez J-c, Leriche A. Descamps M. (2012). New antibacterial microporous CaP materials loaded with phages for prophylactic treatment in bone surgery. *J. Mater. Sci.* 23 2445–2452. 10.1007/s10856-012-4711-6 22802104

[B69] Mocé-Llivina L. Muniesa M. Pimenta-Vale H. Lucena F. Jofre J. (2003). Survival of bacterial indicator species and bacteriophages after thermal treatment of sludge and sewage. *Appl. Environ. Microbiol.* 69 1452–1456. 10.1128/aem.69.3.1452-1456.2003 12620828 PMC150051

[B70] Moghtader F. Eǧri S. Piskin E. (2017). Phages in modified alginate beads. *Artif. Cell. Nanomed. Biotechnol.* 45 357–363. 10.3109/21691401.2016.1153485 27018340

[B71] Mumper R. J. Huffman A. S. Puolakkainen P. A. Bouchard L. S. Gombotz W. R. (1994). Calcium-alginate beads for the oral delivery of transforming growth factor-β1 (TGF-β1): stabilization of TGF-β1 by the addition of polyacrylic acid within acid-treated beads. *J. Control. Release* 30 241–251. 10.1016/0168-3659(94)90030-2

[B72] Murphy J. Mahony J. Bonestroo M. Nauta A. van Sinderen D. (2014). Impact of thermal and biocidal treatments on lactococcal 936-type phages. *Int. Dairy J.* 34 56–61. 10.1016/j.idairyj.2013.06.011

[B73] Nieth A. Verseux C. Barnert S. Süss R. Römer W. (2015a). A first step toward liposome-mediated intracellular bacteriophage therapy. *Expert Opin Drug Deliv.* 12 1411–1424. 10.1517/17425247.2015.1043125 25937143

[B74] Nieth A. Verseux C. Römer W. (2015b). A question of attire: dressing Up bacteriophage therapy for the battle against antibiotic-resistant intracellular bacteria. *Springer Sci. Rev.* 3 1–11. 10.1007/s40362-014-0027-x

[B75] Nilsson A. S. (2019). Pharmacological limitations of phage therapy. *Upsala J. Med. Sci.* 124 218–227. 10.1080/03009734.2019.1688433 31724901 PMC6968538

[B76] Nithya S. Nimal T. R. Baranwal G. Suresh M. K. Anil Kumar V. (2018). Preparation, characterization and efficacy of lysostaphin-chitosan gel against Staphylococcus aureus. *Int. J. Biol. Macromolec.* 110 157–166. 10.1016/j.ijbiomac.2018.01.083 29410001

[B77] O’Flynn G. Coffey A. Fitzgerald G. F. Ross R. P. (2006). The newly isolated lytic bacteriophages st104a and st104b are highly virulent against *Salmonella enterica*. *J. Appl. Microbiol.* 101 251–259. 10.1111/j.1365-2672.2005.02792.x 16834613

[B78] Olsen N. M. C. Thiran E. Hasler T. Vanzieleghem T. Belibasakis G. N. Mahillon J. (2018). Synergistic removal of static and dynamic *Staphylococcus aureus* biofilms by combined treatment with a bacteriophage endolysin and a polysaccharide depolymerase. *Viruses* 10:8. 10.3390/v10080438 PMC6116285 30126174

[B79] Onsea J. Soentjens P. Djebara S. Merabishvili M. Depypere M. Spriet I. (2019). Bacteriophage application for difficult-to-treat musculoskeletal infections: development of a standardized multidisciplinary treatment protocol. *Viruses* 11:891. 10.3390/v11100891 31548497 PMC6832313

[B80] Otero J. García-Rodríguez A. Cano-Sarabia M. Maspoch D. Marcos R. Cortés P. (2019). Biodistribution of liposome-encapsulated bacteriophages and their transcytosis during oral phage therapy. *Front. Microbiol.* 10:689. 10.3389/fmicb.2019.00689 31019499 PMC6458305

[B81] Pastagia M. Euler C. Chahales P. Fuentes-Duculan J. Krueger J. G. Fischetti V. A. (2011). A novel chimeric lysin shows superiority to mupirocin for skin decolonization of methicillin-resistant and -sensitive *Staphylococcus aureus* strains. *Antimicrob. Agents Chemother.* 55 738–744. 10.1128/aac.00890-10 21098252 PMC3028755

[B82] Pennone V. Sanz-Gaitero M. O’Connor P. Coffey A. Jordan K. van Raaij M. J. (2019). Inhibition of *L. monocytogenes* biofilm formation by the amidase domain of the phage vB_LmoS_293 endolysin. *Viruses* 11:722. 10.3390/v11080722 31390848 PMC6723838

[B83] Prasuhn D. E. Jr. Singh P. Strable E. Brown S. Manchester M. Finn M. G. (2008). Plasma clearance of bacteriophage Qbeta particles as a function of surface charge. *J. Am. Chem. Soc.* 130 1328–1334. 10.1021/ja075937f 18177041 PMC2657921

[B84] Puapermpoonsiri U. Ford S. J. van der Walle C. F. (2010). Stabilization of bacteriophage during freeze drying. *Int. J. Pharmaceut.* 389 168–175. 10.1016/j.ijpharm.2010.01.034 20105458

[B85] Puapermpoonsiri U. Spencer J. van der Walle C. F. (2009). A freeze-dried formulation of bacteriophage encapsulated in biodegradable microspheres. *Eur. J. Pharmaceut. Biopharmaceut.* 72 26–33. 10.1016/j.ejpb.2008.12.001 19118627

[B86] Rayment P. Wright P. Hoad C. Ciampi E. Haydock D. Gowland P. (2009). Investigation of alginate beads for gastro-intestinal functionality, Part 1: in vitro characterisation. *Food Hydrocol.* 23 816–822. 10.1016/j.foodhyd.2008.04.011

[B87] Rubalskii E. Ruemke S. Salmoukas C. Aleshkin A. Bochkareva S. Modin E. (2019). Fibrin glue as a local drug-delivery system for bacteriophage PA5. *Sci. Rep.* 9:2091. 10.1038/s41598-018-38318-4 PMC6376040 30765740

[B88] Salalha W. Kuhn J. Dror Y. Zussman E. (2006). Encapsulation of bacteria and viruses in electrospun nanofibres. *Nanotechnology* 17 4675–4681. 10.1088/0957-4484/17/18/025 21727596

[B89] Schmelcher M. Donovan D. M. Loessner M. J. (2012). Bacteriophage endolysins as novel antimicrobials. *Fut. Microbiol.* 7 1147–1171. 10.2217/fmb.12.97 23030422 PMC3563964

[B90] Schuch R. Nelson D. Fischetti V. A. (2002). A bacteriolytic agent that detects and kills *Bacillus anthracis*. *Nature* 418 884–889. 10.1038/nature01026 12192412

[B91] Segale L. Giovannelli L. Mannina P. Pattarino F. (2016). Calcium alginate and calcium alginate-chitosan beads containing celecoxib solubilized in a self-emulsifying phase. *Scientifica* 2016:8. 10.1155/2016/5062706 PMC4834166 27127680

[B92] Shlezinger M. Friedman M. Houri-Haddad Y. Hazan R. Beyth N. (2019). Phages in a thermoreversible sustained-release formulation targeting E. faecalis in vitro and in vivo. *PLoS One.* 14:e0219599. 10.1371/journal.pone.0219599 31291645 PMC6620107

[B93] Silhavy T. J. Kahne D. Walker S. (2010). The bacterial cell envelope. *Cold Spring Harb Perspect Biol.* 2 a000414. 10.1101/cshperspect.a000414 PMC2857177 20452953

[B94] Singla S. Harjai K. Katare O. P. Chhibber S. (2016). Encapsulation of bacteriophage in liposome accentuates its entry in to macrophage and shields it from neutralizing antibodies. *PLoS One* 11:e0153777. 10.1371/journal.pone.0153777 27115154 PMC4846161

[B95] Smidsrød O. Skja°k-Br-— k G. (1990). Alginate as immobilization matrix for cells. *Trends Biotechnol.* 8 71–78. 10.1016/0167-7799(90)90139-o 1366500

[B96] Sulakvelidze A. Kutter E. (2004). “Bacteriophage therapy in humans,” in *Bacteriophages: Biology and Applications*, eds Kutter E. Sulakvelidze A. (Boca Raton, FL: CRC Press), 381–436.

[B97] Thorner J. Emr S. D. Abelson J. N. (2000). “Methods in enzymology,” in *Methods in Enzymology*, Vol. 328 eds Thorner J. Emr S. D. Abelson J. N. (Cambridge, MA:Academic Press), xv–xxxiv.

[B98] Torchilin V. P. (2005). Recent advances with liposomes as pharmaceutical carriers. *Nat. Rev. Drug Discovery* 4 145–160. 10.1038/nrd1632 15688077

[B99] Ventola C. L. (2015). The antibiotic resistance crisis: part 1: causes and threats. *P T.* 40 277–283. 25859123 PMC4378521

[B100] Vinner G. K. Vladisavljević G. T. Clokie M. R. J. Malik D. J. (2017). Microencapsulation of clostridium difficile specific bacteriophages using microfluidic glass capillary devices for colon delivery using pH triggered release. *PLoS One* 12:e0186239. 10.1371/journal.pone.0186239 29023522 PMC5638336

[B101] Vinner K. G. Rezaie-Yazdi Z. Leppanen M. Stapley G. A. Leaper C. M. Malik J. D. (2019). Microencapsulation of *Salmonella*-specific bacteriophage felix O1 using spray-drying in a pH-responsive formulation and direct compression tableting of powders into a solid oral dosage form. *Pharmaceuticals* 12:43. 10.3390/ph12010043 30909381 PMC6469172

[B102] Walsh S. Shah A. Mond J. (2003). Improved pharmacokinetics and reduced antibody reactivity of lysostaphin conjugated to polyethylene glycol. *Antimicrob. Agents Chemother.* 47 554–558. 10.1128/aac.47.2.554-558.2003 12543658 PMC151727

[B103] Wittebole X. De Roock S. Opal S. M. (2014). A historical overview of bacteriophage therapy as an alternative to antibiotics for the treatment of bacterial pathogens. *Virulence* 5 226–235. 10.4161/viru.25991 23973944 PMC3916379

[B104] Wroe J. A. Johnson C. T. García A. J. (2020). Bacteriophage delivering hydrogels reduce biofilm formation in vitro and infection in vivo. *J. Biomed. Mater. Res. Part A.* 108 39–49. 10.1002/jbm.a.36790 31443115 PMC6880309

[B105] Żaczek M. Łusiak-Szelachowska M. Jończyk-Matysiak E. Weber-Dąbrowska B. Międzybrodzki R. Owczarek B. (2016). Antibody production in response to *Staphylococcal* MS-1 phage cocktail in patients undergoing phage therapy. *Front. Microbiol.* 7:1681. 10.3389/fmicb.2016.01681 27822205 PMC5075762

[B106] Zelasko S. Gorski A. Dabrowska K. (2017). Delivering phage therapy per os: benefits and barriers. *Exp. Rev. Anti Infect. Therapy* 15 167–179. 10.1080/14787210.2017.1265447 27885865

[B107] Zhai S. Hansen R. K. Taylor R. Skepper J. N. Sanches R. Slater N. K. (2004). Effect of freezing rates and excipients on the infectivity of a live viral vaccine during lyophilization. *Biotechnol. Prog.* 20 1113–1120. 15296437 10.1021/bp034362x

[B108] Zottola E. A. Marth E. H. (1966). Thermal inactivation of bacteriophages active against lactic *Streptococci*. *J. Dairy Sci.* 49 1338–1342. 10.3168/jds.s0022-0302(66)88091-8 6008383

